# Ultra‐high‐field fMRI insights on insight: Neural correlates of the Aha!‐moment

**DOI:** 10.1002/hbm.24073

**Published:** 2018-04-17

**Authors:** Martin Tik, Ronald Sladky, Caroline Di Bernardi Luft, David Willinger, André Hoffmann, Michael J Banissy, Joydeep Bhattacharya, Christian Windischberger

**Affiliations:** ^1^ MR Center of Excellence, Center for Medical Physics and Biomedical Engineering, Medical University of Vienna Wien Austria; ^2^ Queen Mary University of London School of Biological and Chemical Sciences London United Kingdom; ^3^ Department of Psychology Goldsmiths University of London London United Kingdom

**Keywords:** 7 Tesla fMRI, affect, creativity, dopamine, insight, language processing, learning, RAT, subcortical

## Abstract

Finding creative solutions to difficult problems is a fundamental aspect of human culture and a skill highly needed. However, the exact neural processes underlying creative problem solving remain unclear. Insightful problem solving tasks were shown to be a valid method for investigating one subcomponent of creativity: the Aha!‐moment. Finding insightful solutions during a remote associates task (RAT) was found to elicit specific cortical activity changes. Considering the strong affective components of Aha!‐moments, as manifested in the subjectively experienced feeling of relief following the sudden emergence of the solution of the problem without any conscious forewarning, we hypothesized the subcortical dopaminergic reward network to be critically engaged during Aha. To investigate those subcortical contributions to insight, we employed ultra‐high‐field 7 T fMRI during a German Version of the RAT. During this task, subjects were exposed to word triplets and instructed to find a solution word being associated with all the three given words. They were supposed to press a button as soon as they felt confident about their solution without further revision, allowing us to capture the exact event of Aha!‐moment. Besides the finding on cortical involvement of the left anterior middle temporal gyrus (aMTG), here we showed for the first time robust subcortical activity changes related to insightful problem solving in the bilateral thalamus, hippocampus, and the dopaminergic midbrain comprising ventral tegmental area (VTA), nucleus accumbens (NAcc), and caudate nucleus. These results shed new light on the affective neural mechanisms underlying insightful problem solving.

## 1 INTRODUCTION

Finding creative solutions to difficult problems is a fundamental aspect of human culture. This process often occurs with a unique phenomenal experience, the Aha!‐moment, referring to the moment of transition from being completely dark about the solution to suddenly “seeing” it. This phenomenological experience brings a sense of ease, is intrinsically pleasurable, and accompanied by a feeling of certainty about the solution (Shen, Yuan, Liu, & Luo, 2016; Topolinski & Reber, 2010). One of the first anecdotal evidence of Aha! or Eureka!‐moment was associated with Archimedes, a leading scientist in classical antiquity, who had leapt from his bath and shouted “Eureka! (Greek meaning, “I have found it”) when he suddenly (from his perspective) found out a brilliant solution to a difficult problem (Biello, 2006). Since that time, many scientists were described to have this experience as source for their groundbreaking ideas, among them Carl Friedrich Gauss, Albert Einstein, and Sir Alec Jeffreys. Due to its mystical phenomenology, 20th century psychologists started to get to the bottom of this observation first coined by the famous psychologist Karl Bühler (Bühler, 1907). Since then insightful problem solving has been associated with many different cognitive and affective processes as memory, enforcement learning, and emotion (Kizilirmak, Thuerich, Folta‐Schoofs, Schott, & Richardson‐Klavehn, 2016a; Milivojevic, Vicente‐Grabovetsky, & Doeller, 2015; Shen et al., 2016; Webb, Little, & Cropper, 2017). Despite the importance of the Aha!‐experience in obtaining creative and insightful solutions and the large corpus of behavioral evidence, imaging studies on the brain mechanisms involved in this phenomenon just emerged recently.

A few fMRI and EEG studies exist on insight, reporting cortical areas that include parts of the temporal lobes, especially the superior temporal gyrus, and parts of the prefrontal cortex (Dietrich & Kanso, 2010; Jung‐Beeman et al., 2004). These results indicate higher order cognitive processes as task monitoring and (semantic) retrieval to be at the core of Aha!‐experience, which is in line with psychological models on insight (Knoblich, Ohlsson, & Raney, 2001; MacGregor, Ormerod, & Chronicle, 2001). However, given the fact that the Aha!‐experience is usually associated with an affective state best described in parallel to reward processing (Canestrari, Bianchi, Branchini, Burro, & Savardi, 2017), dopaminergic midbrain and associated brain structures are expected to be involved in this phenomenon as well. Subtle activation changes, not exceeding strict statistical thresholds, were reported in subcortical areas such as bilateral hippocampi, parahippocampal gyri, and anterior and posterior cingulate cortex (Subramaniam, Kounios, Parrish, & Jung‐Beeman, 2009; Zhao et al., 2013). Furthermore, in a recent study, Kizilirmak et al. (2016a) found left hippocampal and parahippocampal activation during insight, though not surviving a strict threshold, and a significant activation in the anterior cingulate cortex (ACC). Additionally, event‐related potentials indicate that the ACC as well as the parahippocampal gyrus are involved in insightful problem solving (Mai, Luo, Wu, & Luo, 2004; Qiu & Zhang, 2008).

Taking into account a strong affective and learning component as part of the insight experience (Cranford & Moss, 2012; Metcalfe, 1986a, 1986b) and the newly drawn link between creativity and dopaminergic activity (Chermahini & Hommel, 2010; Flaherty, 2005; Kulisevsky, Pagonabarraga, & Martinez‐Corral, 2009; Lhommee et al., 2014; Salvi, Bricolo, Franconeri, Kounios, & Beeman, 2015; Schwingenschuh, Katschnig, Saurugg, Ott, & Bhatia, 2010; Zabelina, Colzato, Beeman, & Hommel, 2016), raises the question if the influence of subcortical areas during insight processing was underestimated so far. This assumption becomes even clearer considering the results of a comprehensive psychological study, where the participants had to freely describe their emotional states associated with insight (Shen et al., 2016), and the three main emotions identified with an Aha‐moment are happy, ease, and certainty. 3 Tesla fMRI studies on cortical underpinnings of insight and EEG studies to capture short‐lived phenomena associated with Aha! focused on cognitive components and led to illuminating insights. However, the neural underpinnings of the aforementioned affective component of the Aha!‐moment remains to be unraveled. Therefore, fast high‐resolution imaging techniques in combinations with elaborated insightful problem solving tasks are needed to answer some of the remaining questions on insightful problem solving tackled in this study.

The compound word RAT was used and validated in previous neuroimaging studies as an appropriate instrument to measure insight problem solving (Jung‐Beeman et al., 2004; Sandkuhler & Bhattacharya, 2008). The goal of compound word remote associates tasks is to find a word that makes three compound words with three given stimulus words. The participants in our study were instructed to answer promptly as soon as they feel confident about their solution, without making a strong revision of the found answer, which allows defining the very moment of the subject experiencing an insight or Aha!

Here we investigated insightful problem solving using fMRI at, for the first time, ultra‐high magnetic field (7 T). We applied an optimized acquisition protocol allowing for high spatial resolution required to reveal BOLD signal changes in subcortical structures during insight. More specifically, we employed the RAT at 7 T to acquire functional brain images with high spatial resolution (voxel size = 1.5 × 1.5 × 1 mm^3^) to adequately image subcortical regions, such as the nucleus accumbens, hippocampus, and the dopaminergic midbrain. Thereby we assessed activation in areas involved in the phenomenological aspects of positive effect, the feeling of certainty about a solution found with insight, and the formation of new memories and associations.

## 2 PARTICIPANTS AND METHODS

### 2.1 Study population

Thirty healthy volunteers were recruited for participation in the fMRI study from the general public via flyers and online platforms. One subject (male) had to be excluded after data acquisition due to noncompliance with task instructions, resulting in a final sample of 29 subjects (15f/14m, age mean ± standard deviation [min, max]: 27.7 ± 3.7 [21, 38] years). Standard fMRI exclusion criteria were applied that included neurological or psychiatric abnormalities, claustrophobia, use or abuse of psychotropic substances, the presence of metallic objects on or inside the body that could not be removed before the measurement, implants such as pacemakers, and pregnancy. They were furthermore checked for nonverbal reasoning as a proxy for general intelligence using the adaptive matrices test (Hornke, Etzel, & Rettig, 2003). This test revealed a mean performance of 88% (*SD* = 12%) correctly solved trials in our sample, indicating normal to above average general intelligence in these participants.

### 2.2 fMRI task

The remote associates test (RAT) was shown to be one of the most promising experimental setups to investigate insightful problem solving. In this task, the subject is presented with a sequence of three words (e.g., HOUSE–BARK–APPLE) and instructed to find a fourth word to form an associated compound noun (e.g., TREE). The methodological benefit of this task is that it encompasses a divergent thinking and a convergent thinking task component. Although in the creativity literature the RAT is usually associated with convergent thinking, successful RAT solution requires both divergent and convergent thinking (Koutstaal & Binks, 2015). To obtain the solution word, we often have to suppress those words that are closely associated with the presented words, and instead, search for the word that is remotely associated, thereby requiring a break from our habitual mode of thinking, a key criterion for divergent thinking. Divergent thinking is required as the most salient solution candidates (i.e., those with the closest associations) are supposed to be incorrect to fully qualify as a proper insight solution. However, the RAT solutions are usually unambiguous and, once uncovered, easily reportable, which is ideal for controlled experiments (Dietrich & Kanso, 2010; Salvi et al., 2015). Finding the solution is therefore the convergence towards a definite goal.

**Figure 1:**
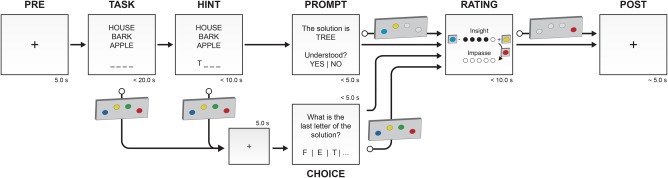
Flow chart of an RAT trial. Each trial consists of 6 phases, with fixed and variable durations. A schematic representation of the response box that was used during the experiments indicates the possibility of button presses to report a solution (during TASK or HINT), to select one of the presented options (PROMPT, CHOICE), and to provide a subjective rating on a discrete scale (RATING) [Color figure can be viewed at http://wileyonlinelibrary.com]

For this study, a German version of the RAT has been developed and implemented in Python 2.7 using pyglet as graphical front end. The flow chart of an experimental trial is shown in Figure 1. Each trial consisted of the following phases: (a) PRE: crosshair at the beginning displayed for 5 s. (b) TASK: word triplet and a series of underscore characters (i.e., “_”) to indicate the length of the correct solution presented for a maximum duration of 20 s. A button press allowed the subject to report that they found a solution. On button press, a crosshair was shown for 5.0 s. (c) HINT: if the subject did not press a button during the 20 s TASK period, the first letter of the solution was revealed as a hint. The maximum length of this phase was 10 s. (4) PROMPT: if the subject did not respond in time, the correct solution was presented for 5 s and the subject had to indicate if they understood the solution or not. (5) CHOICE: if the subject pressed a button during TASK or HINT, they were asked to verify their solution. (6) RATING: at the end of each trial the subject was asked to rate their subjective experience during the trial. On a discrete scale from 0 to 5, they indicated the amount of insight and impasse they experienced during the task. (7) POST: crosshair at the end to ensure a total length of each trial sums up to 60 s. Participants attempted to solve 48 RAT items (randomly selected from the 135 German RAT items) inside the MRI scanner. These items were subdivided into 4 runs (12 RAT items per run).

For both CHOICE and RATING periods, the subject reacted nonverbally. Solution verification and the amount of insight/impasse were done via a button press on a 4‐key keypad. To verify the right solution during CHOICE, we asked for the last letter of the solution word, that is, 3 letters were presented with an additional option for [other]. The subjects had to press the corresponding button on the controller. For the RATING scales (insight/impasse), a 6‐point Likert‐like scale was shown and button 1 (+) and 2 (−) were used to set the amount.

Considering that the list of validated compound word RAT was published in English, we developed a German version of the task, by translating the version adapted by Sandkuhler and Bhattacharya (2008) into German. Although there are other recent German translations of the RAT available (Kizilirmak, Wiegmann, & Richardson‐Klavehn, 2016b; Landmann et al., 2014), no validated tasks were available when we setup the study. Moreover, developing our own translation accounts for some cultural semantic differences between German Standard German and Austrian Standard German. Translating the task into German was an important step, as the test population consisted exclusively of German native‐speakers. To evaluate the translated items before they could be used in the fMRI experiment, one native German speaker assessed these items for strange or uncommon items. Subsequently, a sample of five native German speakers who did not participate in the subsequent fMRI study attempted to solve the items. Based on their evaluation, 135 translated items were chosen. In order to validate if this German version evoked a balanced amount of insight ratings we additionally administered an online version of the paradigm to an additional sample of 163 subjects. Analogous to the English version of the compound word RAT, the German version was supposed to consist of items with varying degrees of difficulty. Also, it should provide items that could be solved either analytically or with a sudden insight. Those two premises were essential to collect control trials for fMRI data analysis and they are demonstrated in the results session. Importantly, the varying degree of difficulty for different items allowed us to collect a number of trials that could be solved with or without hint or were too hard to be solved.

Measurements were performed on a MAGNETOM 7T whole‐body MR scanner (Siemens, Erlangen, DE) at the MR Centre of Excellence, Medical University of Vienna, Austria. For data acquisition a 32‐channel head coil was used with the CMRR multiband EPI sequence (Moeller et al., 2010). The sequence parameters to acquire 508 volumes for each of the four sessions were as follows: repetition time TR = 1.4 s, echo time TE = 23 ms, flip angle α = 62°, 78 slices with a spatial resolution of 1.5 × 1.5 × 1 mm^3^ (slice gap 0.25 mm). Note that using such small voxels sizes increases fMRI sensitivity in ventral brain areas as signal losses from intravoxel dephasing effects due to the presence of field inhomogeneity are strongly reduced (Robinson, Windischberger, Rauscher, & Moser, 2004; Windischberger, Robinson, Rauscher, Barth, & Moser, 2004). Stimuli were shown on a screen mounted at the scanner bore via a video projector. A mirror was used to allow subjects to view the stimuli while lying comfortably inside the MR scanner. Feedback from the subject (i.e., responding to a given task) was recorded by the use of an MR compatible response box (Current Designs, Philadelphia, PA).

All subjects were financially reimbursed for their participation and provided informed written consent. The study protocol was approved by the institutional review board of the Medical University of Vienna. The study was performed in accordance with the Declaration of Helsinki (1964), including current revisions.

### 2.3 Preprocessing and general linear model (GLM) analysis of fMRI data

Data were slice‐timing corrected (FSL) (Sladky et al., 2011), bias‐field corrected (ANTs) (Avants et al., 2011), realigned (FSL), normalized (ANTs), and spatially smoothed with a 3 mm FWHM Gaussian kernel (FSL) using a custom preprocessing pipeline to focus on small subcortical structures. Data analyses were performed in SPM12. For single‐subject (first level) analysis, linear regression was performed at each voxel, using generalized least squares with a global approximate AR(1) autocorrelation model, drift fit with Discrete Cosine Transform basis (128 s cutoff).

Single‐subject GLM analyses were conducted for all four sessions and included boxcar functions to model longer task periods (i.e., UNSOLVED TASK, SOLVED TASK with or without insight and with or without hint, HINT, CHOICE, PROMPT, RATING) and stick functions for events (i.e., EVENT of found solution with high or low insight and a timeout event for UNSOLVED). As criterion for an item solved with high insight versus low insight, we calculated mean splits for each run per subject and accounted insights above individual means as solved with insight and trials solved below mean as solved without insight. This way we guaranteed a balanced amount of trials per condition and accounted for inter‐ and intraindividual differences, for example, learning effects and different concepts on scoring on a Likert scale (Csikszentmihalyi & Larson, 2014) over the course of the experiment. Both types of regressors were convolved with SPM's canonical HRF. Additionally, realignment parameter estimates were added as nuisance regressors to model movement‐related variance not accounted for during preprocessing.

Resulting contrast maps from single‐subject GLM analyses were used for group analysis as implemented in SPM12, that is, linear regression was performed at each voxel, using generalized least squares with a global repeated measures correlation model.

**Figure 2:**
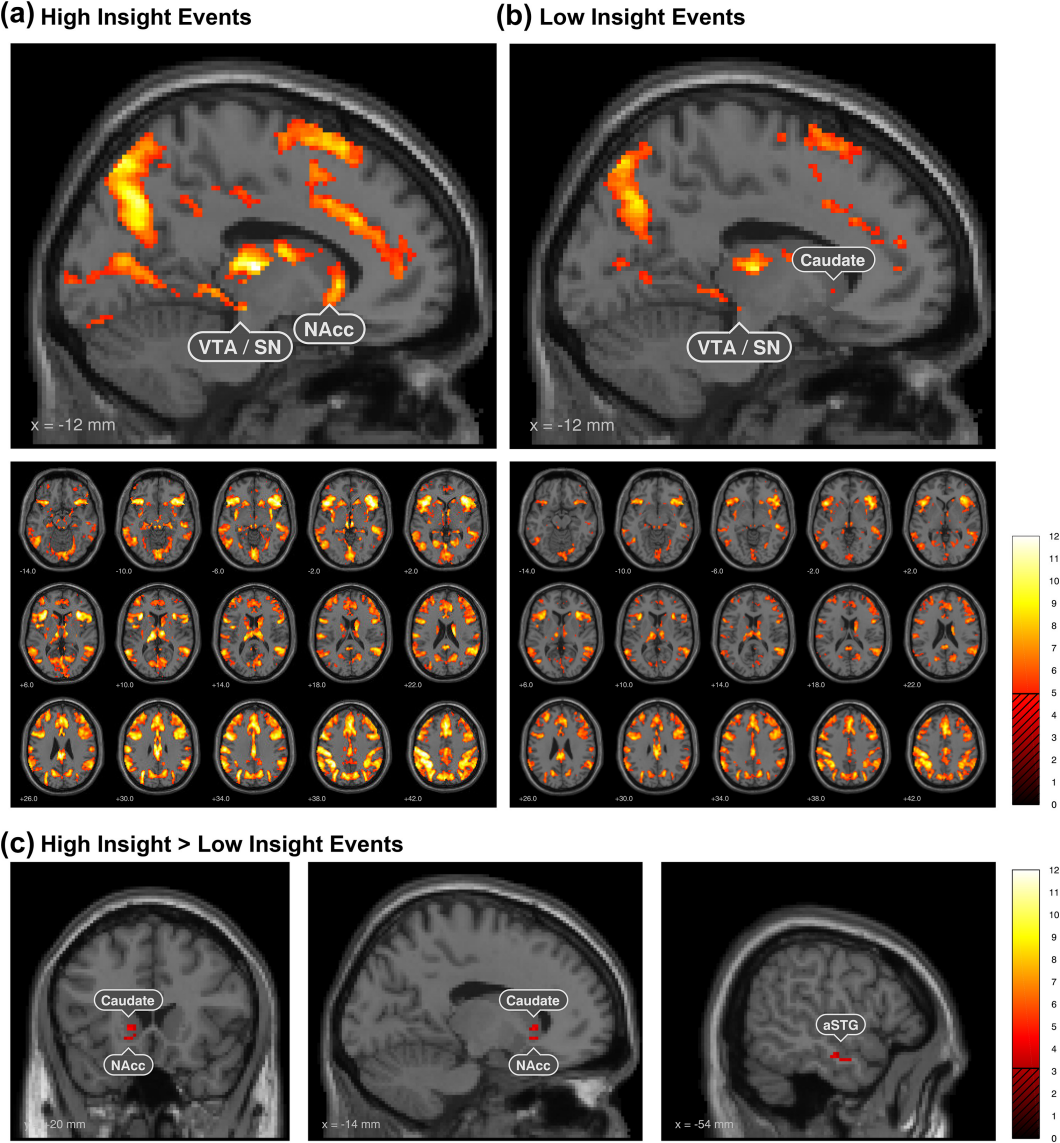
Neural correlates of Insight. Significant brain activation changes (*p* < .05 FWE whole‐brain) when the solution was found with a low feeling of Insight, that is, more analytical solutions (RIGHT) including areas associated with semantic memory retrieval and during Aha!‐moments, that is, solutions with a high amount of Insight (LEFT), featuring the same areas and additional stronger VTA and NAcc activations. Contrast of trials solved with high versus low subjective Insight rating (BOTTOM, *p* < .05 cluster‐level) shows the anterior superior temporal sulcus/gyrus (Jung‐Beeman et al., 2004) and importantly highlights the nucleus accumbens (NAcc) as the subcortical core region of the Aha!‐moment [Color figure can be viewed at http://wileyonlinelibrary.com]

To map brain‐activation during single task conditions, we calculated *t* maps with a threshold of *p* < .05 with whole‐brain FWE correction (Figure 2 High Insight, Low Insight).

**Figure 3:**
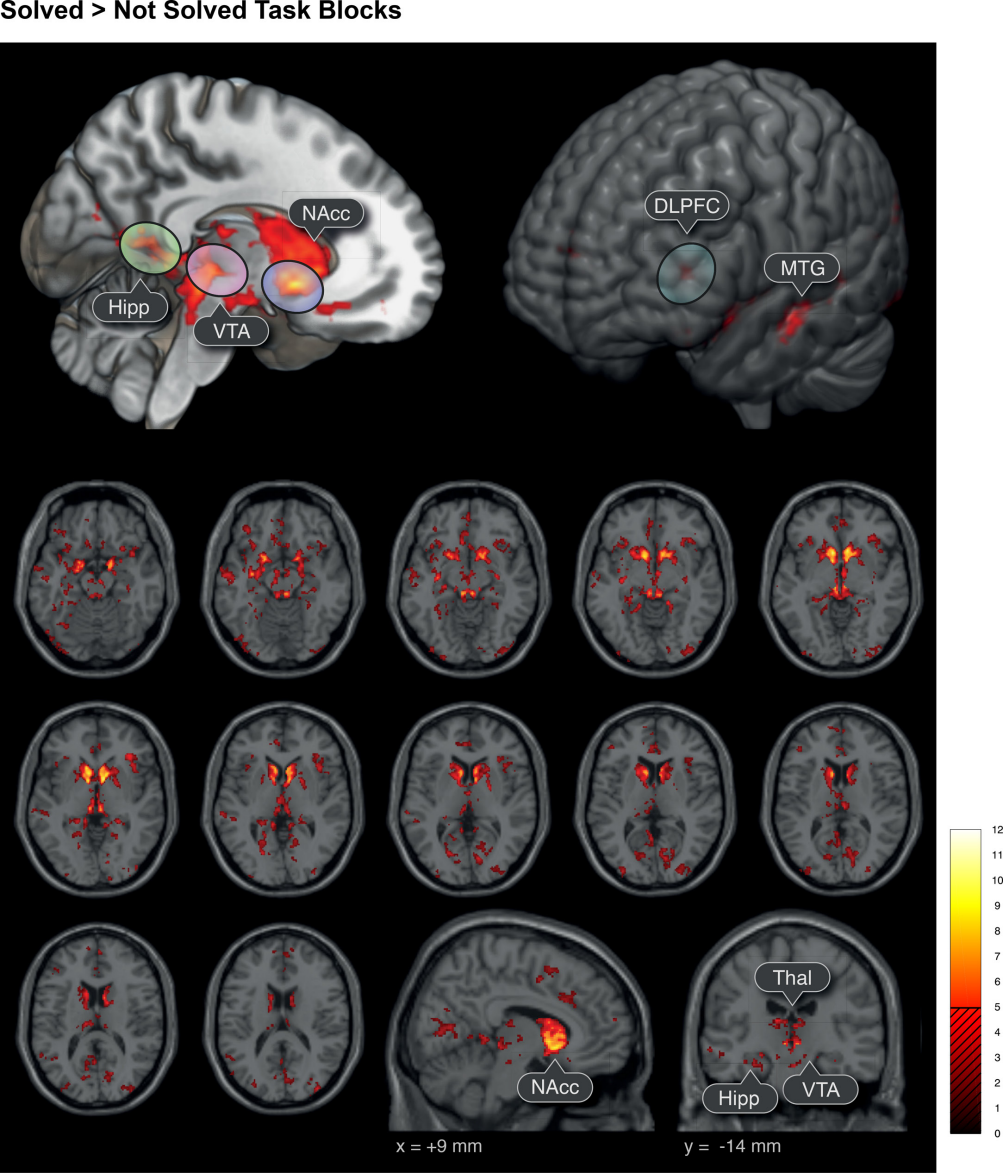
Brain activation during TASK related to successful problem‐solving. Significant brain activations for the whole TASK period (i.e., the total length of the RAT task block) when solved compared to unsolved trials. The threshold of the *t* statistics was set to *p* < .05, cluster‐wise FWE correction (initial cluster defining threshold *p* < .001). Hipp, and thalamic regions, MTG, IPL seem to be related to successful solution of a language task and reinforcement learning. Highest activation changes were observed in subcortical and cortical dopaminergic regions (i.e., NAcc, VTA) and DLPFC leading to the hypothesis that insightful problem solving is highly dependent on different dopaminergic pathways (Boot et al., 2017) [Color figure can be viewed at http://wileyonlinelibrary.com]

To detect differences between conditions (Figure 2 High Insight > Low Insight, Figure 3 Solved > Not Solved) we calculated *t* statistics *p* < .05 cluster‐wise FWE correction with an initial cluster defining threshold of *p* < .001.

For anatomical labeling of activation patterns, we used the TT_Daemon atlas in AFNI (whereami function). To perform region of interest analysis, we used marsbar (Brett, Anton, Valabregue, & Poline, 2002) to extract mean beta values from bilateral NAcc, hippocampus, and VTA separately for solved with insight, solved without insight and unsolved trials. Post‐hoc *t* tests on ROI results were calculated using MATLAB.

### 2.4 Dynamic causal modeling (DCM)

Dynamic causal modeling (DCM) is a well‐established model selection procedure (Friston, Harrison, & Penny, 2003) that is used to identify optimal causal models from an *a priori* defined model space within a fully Bayesian framework. DCM12 (SPM12, build 7134) was used for effective connectivity analysis. Motivated by our findings for task and event condition, detrended time courses of the left DLPFC, NAcc, posterior Hipp, and SN/VTA (anatomical masks that were also used in the VOI analysis) were extracted for each participant using SPM's volume of interest (VOI) extraction batch script based on a single‐subject significance threshold *p* < .05 (first eigenvariate used as summary statistic, adjusted for effect of interest).

In all models, the RAT task blocks were used as driving input for the DLPFC. Bidirectional connections between DLPFC, hippocampus (Hipp), nucleus accumbens (NAcc), and ventral tegmental area (VTA) were modeled. Given that there is no clear *a priori* assumption on the effective connectivity between the VOIs, we created a model space that comprised permutations of all possible bidirectional connections between these regions, that is, 2^2*3^ = 64 different models. On all connections, the events for solution with high insight, solution with low insight, and no solution were modeled as modulators to assess how these conditions alter the effective connectivity. This model space was the basis for a random effects Bayesian model averaging (BMA) to determine a group average of the connectivity parameter estimates.

## 3 RESULTS

### 3.1 Behavioral data

We intended to design a task with a set of compound remote associate problems where, on average, about half of the problems would be solved. As shown in Supporting Information, Table S1, we succeeded to create a translation comparable to (Sandkuhler & Bhattacharya, 2008), with problems that were easy, medium, and hard to solve as tested on an independent sample outside the scanner.

This was transferable on inside scanner behavioral data (Supporting Information, Table S2) where on average, participants proposed solutions for 58% (SD: 49%) of the 48 trials. Out of these answered trials, 71% were correct. The first run evoked average insight ratings of 2.61 (*SD* = 1.42), the second run 2.72 (*SD* = 1.39), the third run 2.87 (*SD* = 1.35), and the fourth 3.02 (*SD* = 1.42). There was no significant correlation between run and insight rating (two‐tailed rho = .179, *p* = .054), indicating no significant training effect. A detailed overview of mean insight rating per run and subject is depicted in Supporting Information, Table S 3.

Behavioral data outside the scanner revealed a mean probability of solving items of 53% (*SD* = 23%, min = 8%, max = 96%). 74% of the items were solved correctly, that is, by the predefined solution, and 26% were solved with an alternative solution. Out of correctly solved items subjects showed a mean insight rating of 1.85 (*SD* = .60, min = 0, max = 4), where 0 means no insight and 5 means highest imaginable insight experience. The probability to solve an item (*r* = −.013, *p* = .879) did not correlate with insight ratings.

### 3.2 fMRI results

Based on the subjective mean split per run and subject insight ratings, successful trials (i.e., trials with solutions) with a rating below individual mean per run were classified as low insight solutions and those with a rating above the mean as high insight solutions. Out of these data *event*, regressors were used to model brain activity changes at the moment of the behavioral response (i.e., button press) indicating the high insight versus low insight condition. The regressors for *task* included the whole period in which the participants were trying to solve the problems and were differentiated into trials that were solved with low insight, with high insight, or not solved.

#### 3.2.1 Event

While task activations for low insight and high insight trials both included the bilateral inferior frontal gyrus (IFG), insular cortex, dorso‐medial prefrontal cortex (DMPFC), precuneus VTA, hippocampus, striatum, and thalamus, the activations for high insight revealed additionally stronger activations in large parts of the striatum most prominent the nucleus accumbens (right peak: 10, 11, −1 [MNI], *T*_peak_ = 10.08, *p*_FWEc_ < .001, left peak: −8, 5, −2 [MNI], *T*_peak_ = 9.96, *p*_FWEc_ < 0.001), and the VTA.

The regions identified from the contrast of high insight versus low insight (Figure 2, bottom) were covering parts of the anterior superior temporal sulcus (aSTS; peak: −51, −10, −20 mm [MNI], *T*_peak_ = 4.53, *p*_FWEc_ = .026), NAcc (peak: −6, 14, −4 mm [MNI], *T*_peak_ = 3.70, *p*_FWEc_ = .005) and caudate nucleus (peak: −14, 20, 0 mm [MNI], *T*_peak_ = 4.53, *p*_FWEc_ = .005).

#### 3.2.2 Task

Comparison of task activity during trials, that is, ongoing problem solving, solved versus unsolved revealed three distinct clusters on the cortex (Figure 3, top right): one cluster located around the middle temporal gyrus (MTG), a second around the dorsolateral prefrontal cortex (DLPFC), and a third cluster in the anterior cingulate cortex (ACC). Additionally, we found a widespread subcortical network to be activated during the task consisting of the thalamus (Thal), hippocampus (Hipp), ventral tegmental area (VTA), and nucleus accumbens (NAcc) (Figure 3, top left).

To better specify most relevant subcortical nuclei, an additional analysis was performed on event contrasts using anatomical masks for nucleus accumbens (AAL atlas) and VTA (Talairach atlas transformed to MNI space).

**Figure 4:**
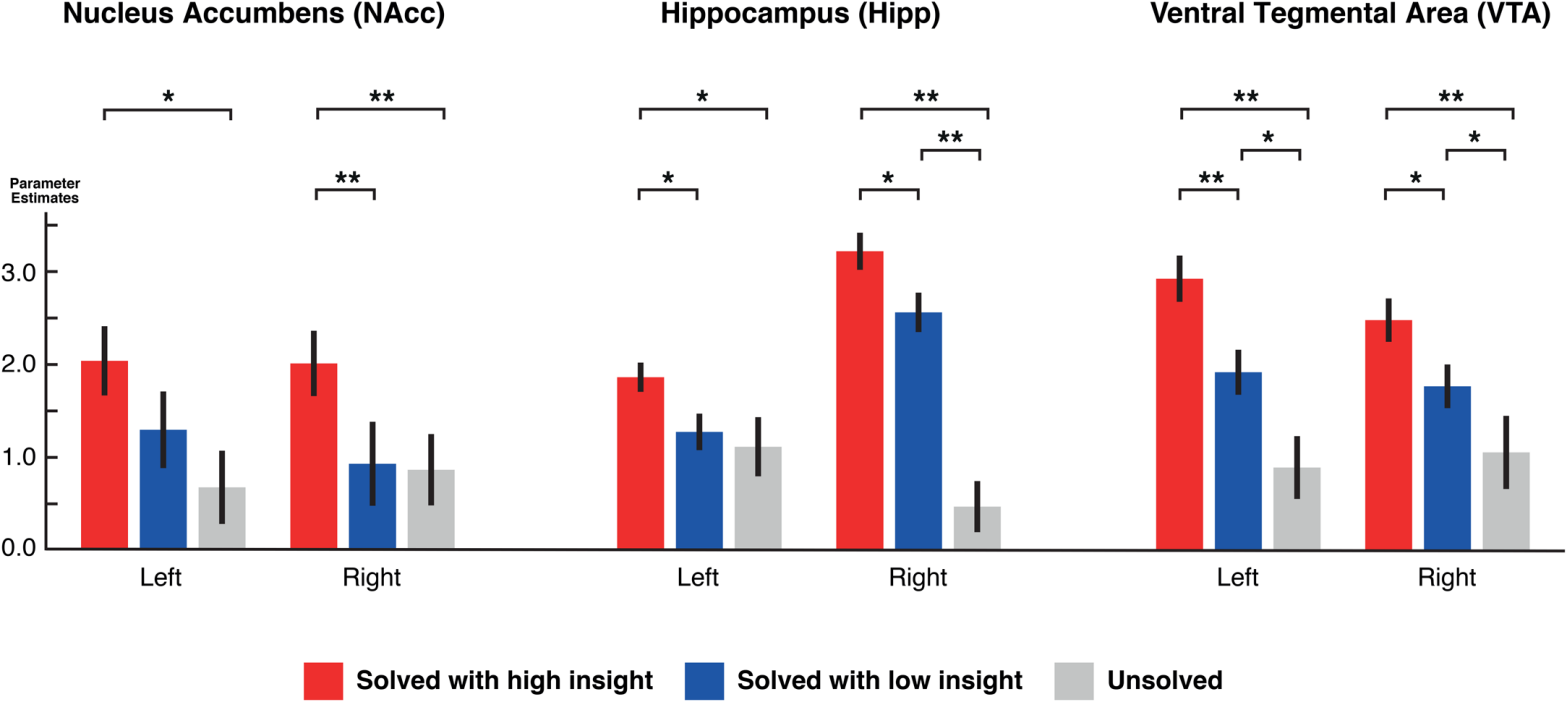
Condition‐dependent activation of subcortical structures. Nucleus accumbens, hippocampus, and VTA activations were significantly stronger for insightfully solved trials. ROI definitions for bilateral NAcc, ventral tegmental area were based on *a priori* information from anatomical atlases, posterior hippocampus ROI is based on group statistics. *p < .05, **p < .01 in *t* test [Color figure can be viewed at http://wileyonlinelibrary.com]

Activation increase in the left nucleus accumbens (NAcc) was associated with insightful problem solving as compared to unsolved trials (*p* < .05) and in the right NAcc as compared to noninsightful and unsolved trials (*p* < .01) (Figure 4, first column).

Given that only the posterior part of hippocampus showed relevant activation in the whole‐brain analysis, we used a functionally defined brain mask based on the significant group activation, to confirm with the approach used in current literature relevant for the study of insight effects (Milivojevic et al., 2015). We found more activation in solution events with insight compared to non‐insight solution (*p* < .05) and no solution (*p* < .05) in the left posterior hippocampus and solved with insight versus without insight (*p* < .05) and insight vs. not solved (*p* < .01) in the right hemisphere (Figure 4, second column).

Bilateral VTA was significantly more active for solutions with insight compared to unsolved trials (*p* < .01) and solved with insight versus without insight (*p* < .01, left hemisphere; *p* < .05, right hemisphere) (Figure 4, third column).

### 3.3 Dynamic causal modeling

**Figure 5:**
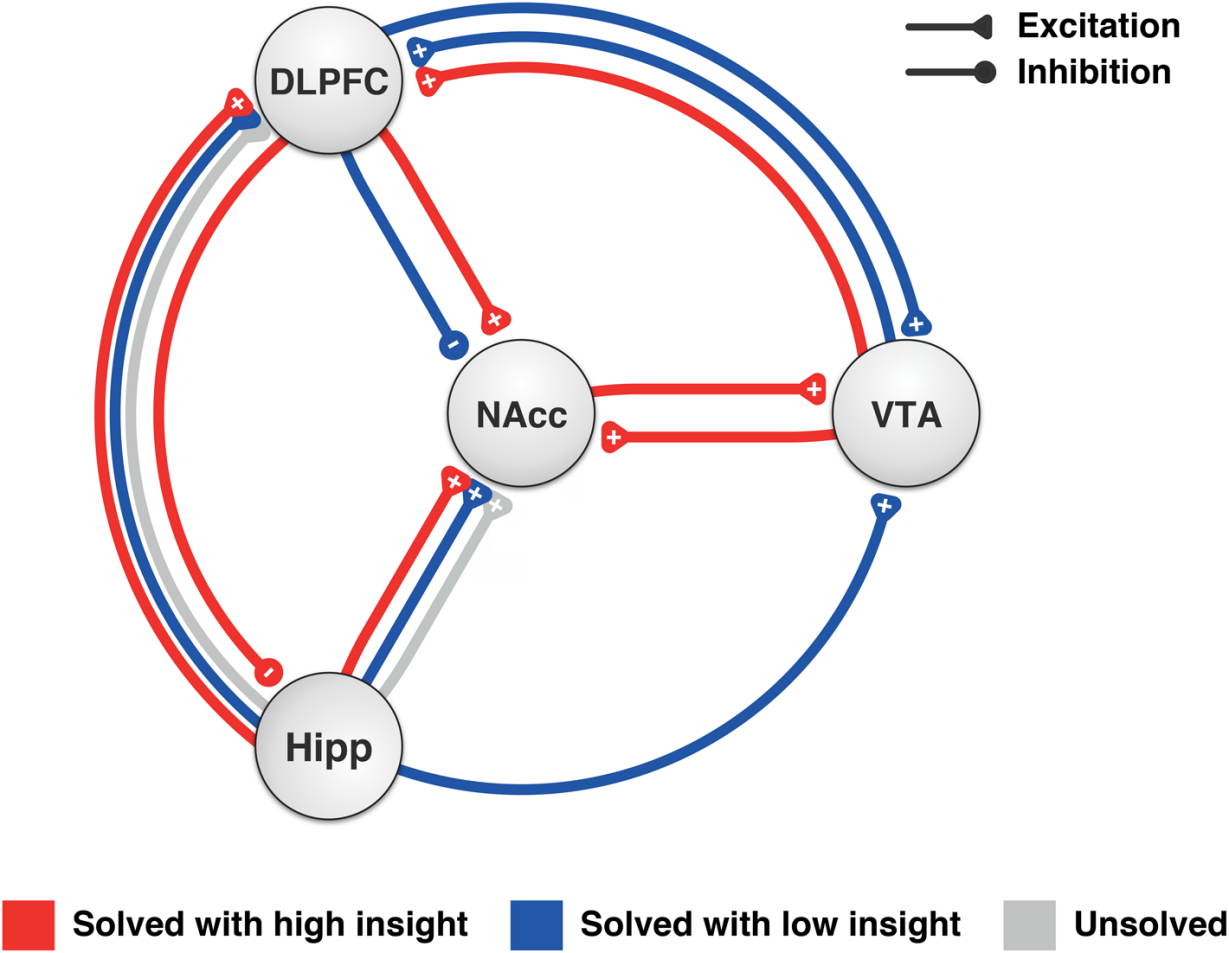
Condition‐dependent effective connectivity of subcortical structures. Most importantly, DLPFC connectivity was only positive during high insight and negative for low insight moments. Additionally, high insight was associated with significantly positive VTA to NAcc forward (+0.072 ± 0.136) and backward connectivity (+0.072 ± 0.134). Bayesian model averaging group results of intrinsic connectivity plus modulation, *p* < .05 [Color figure can be viewed at http://wileyonlinelibrary.com]

Investigating effective connectivity differences between the three possible trial outcomes, we analyzed the sum of intrinsic connectivity (*A*‐matrix) and its modulation by the respective conditions (*B*‐matrix) (Figure 5). We observed that DLPFC connectivity was only positive during high insight moments (+0.095 ±0.121) and negative for low insight moments (−0.051 ±0.121). At the same time, we observed only for high insight, significantly positive VTA to NAcc forward (+0.072 ±0.136) and backward connectivity (+0.072 ±0.134). VTA to DLPFC connectivity was also positive for high (+0.065 ±0.133) and low insight (+0.088 ±0.133), yet not for no solution. Low insight was also characterized by VTA upregulation by the DLPFC (+0.067 ±0.118) and Hippocampus (+0.074 ±0.136), which was not found in the other conditions. Positive connections from Hippocampus to DLPFC and NAcc for all conditions. Finally, the Hippocampus was inhibited during insight moments by the DLPFC (−0.042 ±0.110).

## 4 DISCUSSION

In this study, we focused on revealing brain structures involved during Aha‐moments, and therefore used ultra‐high‐field fMRI at 7 T and a fast, multiband‐accelerated sequence to assess brain activity while participants were solving remote associate task (RAT) problems. A creative endeavor requires divergent thinking (Guilford, 1967; Torrance, 1968) as well as convergent thinking (Dietrich & Kanso, 2010). While common tests restrain creative performance to divergent thinking, for exmaple, the Torrance Test of Creative Thinking (Torrance, 1968) or the Alternative Uses Test (Guilford, 1967), the RAT is designed as a paradigm to measure both convergent and divergent thinking. While some of the problems might be solved analytically, insight trials let the subject experience a sudden jump to a solution experienced as pleasurable Aha!‐moment.

The increased signal‐to‐noise‐ratio (SNR) at ultra‐high magnetic field strengths (Sladky et al., 2013) allowed us to find robust effects in a number of cortical and subcortical areas that are particularly related to a higher level of insight (Figure 2). Thereby we are (a) corroborating former research linking insight to aSMG and hippocampus and (b) for the first time highlighting subcortical structures of the dopaminergic pathway, particularly the NAcc as a critical hub linked to this very moment of creative insight (Aha!). Interestingly, we found that the nucleus accumbens (NAcc) showed higher BOLD response during the solved versus not solved trials, and was also modulated by insight: higher insights evoked increased NAcc activations as compared to lower insights. For the EVENT of insight, the NAcc was the most prominent area specifying this phenomenon. NAcc has been implicated in reward processing as it responds to pleasant stimuli or positive reinforcement (Abler, Walter, Erk, Kammerer, & Spitzer, 2006; Sabatinelli, Bradley, Lang, Costa, & Versace, 2007), however, its functions are not restricted to the processing of primary rewards alone (Salamone, Correa, Mingote, & Weber, 2005). This brain structure receives inputs from hippocampus, amygdala, and prefrontal cortex (e.g., orbitofrontal, medial prefrontal, and ACC) and sends outputs to basal ganglia, dorsal thalamus, substantia nigra (SN), ventral tegmental area (VTA), and the reticular formation (Floresco, Blaha, Yang, & Phillips, 2001; Haber & McFarland, 1999). Resting‐state functional connectivity (Cauda et al., 2011) revealed that the NAcc is functionally connected to the orbitofrontal and prefrontal cortex, globus pallidus, thalamus, midbrain, amygdala, and insula. These structural connections place the NAcc in a good position to functionally integrate processes within subcortical and cortical regions. The connection between NAcc, hippocampus, and medial prefrontal cortex (see below), has the potential to explain the effects of positive mood on insight (Isen, Daubman, & Nowicki, 1987; Subramaniam et al., 2009). Increased nucleus accumbens activation as seen in our study may reflect the sudden jump to a solution candidate accompanied by a moment of relief, ease, joy, and confidence commonly referred to as Aha!‐moment. Supposing that the last phases of insight processing follows a reward like pattern, a rewarding process during insightful problem solving, leads to reinforced learning (conditioning) for insightful solutions resulting in memory consolidation as reflected by increased hippocampal activity. Another area specific to higher insight was the head of the caudate nucleus. Boot, Baas, van Gaal, Cools, and Dreu (2017) propose the striatal pathway to be involved in cognitive flexibility, including perspective switching, divergent thinking, broad attention, and facilitated access to remote associations. Stronger activations in striatal areas associated with insight therefore strongly correspond with the task demands of the RAT and go in line with the proposed model by Boot et al. (2017) of dopaminergic pathways to be involved in different demands of creative thinking. Dopaminergic midbrain structures, such as the VTA and substantia nigra, have recently been linked with the encoding of the expected certainty about a desired outcome (Schwartenbeck, FitzGerald, Mathys, Dolan, & Friston, 2014). While they found an effect related to the estimation of precision of an anticipated future reward, it did not relate to the respective value of this reward. In this study, we found that activation in the VTA was strongly associated with finding solutions (Figure 3), and showed heightened activity during highly insightful trials (Figure 2), which corresponds to the first person phenomenology of certainty that is usually associated with insight moments.

As already discussed by Kounios and Beeman (2014) with EEG and fMRI, the middle temporal cortex is an important cortical hub for insightful problem solving. We were able to extend this finding, showing that the left anterior MTG/STS shows heightened activation for stronger insight solutions. The anterior MTG is involved in phoneme perception (Du, Buchsbaum, Grady, & Alain, 2014). Its involvement in insight during the solution of a verbal jigsaw might represent increased phonemic search as strategy to solve the item. An alternative interpretation is that of Jung‐Beeman (2005), which suggests that temporal areas would be appropriate for integration with unusual or unexpected words. Critically, the anterior MTG/STS remains higher activated for insightful problem solving, which might be indicative for the cognitive functions associated with this brain area to be generally involved in more insightful problem solving. As stated in the *verbal overshadowing theory* cf. Chein and Weisberg (2014), insight will only take place unconsciously and nonverbally eluding the individuals cognitive control. However, being exposed to irrelevant speech during insight problems increased performance (Ball, Marsh, Litchfield, Cook, & Booth, 2015), suggesting that pure phonemic search during RAT might potentially have led to facilitated insight in our study.

The hippocampus has already been linked to insight in previous fMRI studies (Kizilirmak et al., 2016a; Luo & Niki, 2003; Zhao, Zhou, Xu, Fan, & Han, 2014). However, previous studies did not let participants find the solution to word riddles on their own but exposed them to the correct answers with one conventional answer and one novel solution possibility. Therefore it is not clear if this contrast reflects insight in given solution candidates rather than self‐generated, creative solutions. The hippocampus plays a central role in memory consolidation and retrieval. It has been known for a long time that animals (Epstein, Kirshnit, Lanza, & Rubin, 1984) and humans (Auble, Franks, & Soraci, 1979) undergoing problem‐solving tasks, show improved memory for content that was associated with an insight moment. Luo and Niki (2003) were the first to show activation in the right hippocampus (not exceeding FWE threshold) in an fMRI experiment when subjects performed an insightful problem‐solving task (i.e., Japanese riddles) and linked them with the formation of novel associations and breaking of mental fixations. A very recent study on narrative comprehension was able to demonstrate that activation in the posterior part of the hippocampus is strongly associated with the reorganization of memories after an insight, in terms of integrating life‐like events to coherent narratives (Milivojevic et al., 2015). Focusing their analysis on the posterior section of the hippocampus, revealed stronger activation for solution events with insight versus unsolved trials and those solved without insight (left hemisphere: not significant). The present findings support the importance of hippocampal function in the integration and reorganization of associations, particularly those of high novelty that are associated with insight moments.

Neural correlates of solved trials were generally associated with—besides the already discussed areas—the bilateral IFG and insular cortex, inferior parietal lobules (IPL), precuneus, and dorsomedial PFC (DMPFC), especially if the trials were solved with insight, which are all core elements of the semantic memory network. These findings specifically depict what is unique to the solution of linguistic puzzles and similar activation patterns were found in association with metaphor generation (Beaty, Silvia, & Benedek, 2017). Binder and Desai (2011) summarize that the IFG and DMPFC are associated with goal‐direction and selective memory retrieval. The role of semantic memory retrieval in creativity was currently stressed by Benedek et al. (2017). The IPL is in the model of Binder and Desai (2011) is moreover associated with the storage of abstract semantic knowledge, while the precuneus is speculated to build a nexus between the semantic memory system and the hippocampus network associated with episodic memory.

Activation patterns to the event of insight (Figure 2, bottom) not only extend former findings (Jung‐Beeman et al., 2004; Sandkuhler & Bhattacharya, 2008; Subramaniam et al., 2009; Zhao et al., 2014) for temporal lobe involvement in RAT to the left hemisphere, but also extend activation patterns to task‐related motivational and affective subcortical areas. This is in accordance to the studies mentioned above (Jung‐Beeman et al., 2004; Kounios et al., 2006), along with subcortical areas such as bilateral amygdala. The latter authors mentioned that the signal on subcortical regions was low, which could have caused some effects to be undetected, especially in regions near the temporal pole and orbitofrontal areas, which are more prone to magnetic susceptibility artifacts.

The activation in pre‐SMA and ACC for high and low insight events, which were even higher for high insight events, can be related to semantic coherence judgments. Using a similar version of the RAT, Ilg et al. (2007) demonstrated that intuitive impressions of semantic coherence are associated with activation in these anterior midline structures. Explicit coherence judgments, however, were lateral and posterior within the inferior parietal lobule and right superior temporal lobe. As already mentioned, our results show robust activation in bilateral IPL for insight solution events.

Comparing the activation during solved trials (whole period in which the participants were trying to solve the problems) to trials not solved revealed three distinct cortical clusters (Figure 3, top): one cluster located around DLPFC, a second cluster in the MTG and a last cluster in the subgenual ACC. While the DLPFC is involved in goal selection (Feil et al., 2010), the middle part of the MTG is an associative area that plays an important role in the representation of abstract semantic knowledge (Binder & Desai, 2011). Medial prefrontal activation is associated with monitoring brain areas for conflicting action tendencies (Botvinick, Cohen, & Carter, 2004), performance monitoring or the evaluation/processing of the solutions (Anderson, Anderson, Ferris, Fincham, & Jung, 2009). Kounios et al. (2006) found heightened activation of the ACC prior to RAT problem presentation for trials that were followed by insight solutions and propose that the ACC's role in problem solving is detection of conflicting solution strategies. According to their view, a highly activated ACC during problem presentation allows detection of nondominant solution candidates. During solution of the task in comparison to nonsolution trials, additional areas of the dopaminergic midbrain including thalamic pathways, VTA, and substantia nigra (SN) as well as the striatum, especially the NAcc and the posterior hippocampus were highly engaged. This stresses the role of a positive reinforcement circuit related to moments of success as mentioned above.

In this study, we have shown that structures of the dopaminergic midbrain are associated with solutions per se and especially with highly insightful solutions. By means of investigating the association between task solution and insightful problem solving in a large independent sample, we showed that there is no relationship between the difficulty of the items and Aha!‐ratings. These results suggest that there is a certain degree of specificity to the insight rating which is not overlapping with pure solution of an item. In addition to the robust replication of activations in cortical regions reported in previous studies, we have established herein an association between insightful problem solving to subcortical structures. Aha!‐moments are characterized by hyperactivation in (a) nucleus accumbens, which has been shown to be involved in the feeling of relief, ease, and joy, (b) VTA, which is related to the encoding of certainty about a decision, (c) the posterior hippocampus, responsible for memory reorganization following an insight, and (d) aSTS/STG associated coarse semantic coding. Solution‐related task processing periods induced stronger activation in (a) regions that are related with implicit judgment of semantic coherence and (b) nucleus accumbens and other structures of dopaminergic midbrain, indicating elevated mood compared to unsuccessful trials as well as (c) the posterior hippocampus, responsible for memory reorganization following a moment of success. Our results thus suggest that the Aha!‐event is a formative situation that goes along with learning processes and increased involvement in creating solutions. We suppose that the interplay between VTA, NAcc, Hippocampus, and aSTS/STG stresses the Aha!‐Moment as a higher cognitive process not purely consisting of affective and rewarding components.

As those structures are part of a dopaminergic pathway, associated with reinforcement, we suggest the Aha!‐Moment as a special form of fast retrieval, combination, and encoding process. Future research is needed to specify the exact network modulations that underlie the Aha!‐Moment in this regard.

## 5 LIMITATIONS

Limitations of this work include the inability to modulate the temporal evolution of the Aha!‐moment. For this purpose, a further combined fMRI/EEG experiment could further bring together evidence from methods with high temporal and spatial resolution. Furthermore, it remains unclear if significant differences between high and low insight reflected a pure affective epiphenomenon of insight as we could not demonstrate a causal relationship. Converging evidence from pharmacological studies and combined TMS/fMRI studies targeting the DLPFC as an effective cortical hub (Tik et al., 2017a, 2017b) could be implemented in the future.

## Supporting information

Additional Supporting Information may be found online in the supporting information tab for this article.

Supporting Information
