# Supplementary material for: Ultra‐high‐field fMRI insights on insight: Neural correlates of the Aha!‐moment
Source: Hum Brain Mapp. 2018 Apr 17;39(8):3241–52. doi: 10.1002/hbm.24073 (PMC6055807; doi:10.1002/hbm.24073)
Supplement: Supplementary file 1 — Supporting Information [file HBM-39-3241-s001.docx]

# Supplementary material

Supplementary Table S 1. Set of compound remote associates administered outside the scanner on an independent sample (n=163), percentage of solved trials, mean solution times.

| Word 1 | Word 2 | Word 3 | Item | Solved% | Reaction Time Mean | Reaction Time SD | N |
| --- | --- | --- | --- | --- | --- | --- | --- |
| Bügeleisen | Schiff | Motor | Dampf | 96 | 12.43 | 7.55 | 24 |
| Teppich | Alarmstufe | Kreuz | Rot | 96 | 14.69 | 9.09 | 25 |
| Nacht | Schreibtisch | Löten | Lampe | 94 | 14.60 | 7.27 | 31 |
| Flocke | Mobil | Fall | Schnee | 94 | 9.65 | 6.39 | 31 |
| Park | Note | Konto | Bank | 93 | 13.30 | 8.12 | 28 |
| Immer | Gras | Smaragd | Grün | 93 | 7.97 | 6.37 | 40 |
| Nummer | Anruf | Buch | Telefon | 93 | 12.67 | 7.64 | 29 |
| Wasser | Mine | Streuer | Salz | 92 | 10.11 | 5.83 | 25 |
| Hirn | Schlag | Mund | tot | 92 | 16.19 | 8.08 | 26 |
| Stopp | Tasche | Turm | Uhr | 92 | 15.53 | 9.18 | 53 |
| Klick | Falle | Feld | Maus | 90 | 14.18 | 7.75 | 29 |
| Spazieren | Jahr | Rund | Gang | 89 | 11.08 | 8.21 | 18 |
| Traum | Licht | Anbruch | Tag | 89 | 12.43 | 7.38 | 37 |
| Schleuse | Einfahrt | Wart | Tor | 89 | 14.94 | 7.62 | 35 |
| Schwein | Fahne | Strom | Mast | 86 | 16.08 | 7.30 | 29 |
| Leiter | Einkauf | Last | Wagen | 86 | 18.01 | 6.71 | 21 |
| Ziel | Linig | Weg | gerade | 85 | 15.88 | 7.06 | 27 |
| Lounge | Party | Kleid | Cocktail | 84 | 13.21 | 7.92 | 25 |
| Punkt | Liegen | Schnitt | Wunde | 84 | 5.88 | 4.37 | 25 |
| Sandwich | Haus | Golf | Club | 83 | 19.70 | 7.80 | 29 |
| Mittag | Wirkung | Frage | Nach | 81 | 19.14 | 7.69 | 32 |
| Kamerad | Lehrer | Hoch | Schule | 81 | 15.60 | 6.73 | 31 |
| Tuch | Tennis | Stamm | Tisch | 81 | 9.86 | 4.07 | 27 |
| Maß | Wurm | Video | Band | 78 | 12.07 | 9.23 | 32 |
| Sicherheit | Kissen | Haar | Nadel | 78 | 18.55 | 8.07 | 27 |
| Finger | Ohr | Straße | Ring | 78 | 13.33 | 7.42 | 23 |
| Notiz | Haus | Eis | Block | 77 | 11.64 | 7.07 | 26 |
| Wurm | Regal | Zeichen | Buch | 77 | 15.02 | 7.73 | 26 |
| Tasche | Transfer | Strafe | Geld | 76 | 14.27 | 8.43 | 34 |
| Hart | Treiben | Fällen | Holz | 74 | 16.13 | 7.34 | 35 |
| Fall | Gabe | Wahl | Aus | 73 | 18.70 | 7.65 | 26 |
| Politur | Finger | Kopf | Nagel | 72 | 16.50 | 7.27 | 25 |
| Brand | Regen | Viertel | Wald | 70 | 17.25 | 8.54 | 30 |
| Zahn | Haare | Schuh | Bürste | 69 | 12.39 | 7.63 | 32 |
| Pelz | Erde | Tasche | Mantel | 69 | 14.28 | 7.71 | 35 |
| Bau | Waschen | Schreiben | Maschine | 69 | 17.88 | 8.85 | 26 |
| Haupt | Kehrer | Laterne | Straße | 69 | 17.08 | 7.11 | 32 |
| Durch | Auf | Bus | Fahrt | 67 | 10.31 | 5.61 | 27 |
| Feuer | Sommer | Platz | Lager | 67 | 15.46 | 8.46 | 36 |
| Arm | Wäsche | Lage | Unter | 67 | 21.27 | 6.38 | 42 |
| Klappe | Nahrung | Pause | Baby | 66 | 13.19 | 8.06 | 38 |
| Pause | Bohne | Schwarz | Kaffee | 66 | 14.87 | 8.64 | 32 |
| Messer | Kuchen | Cottage | Käse | 65 | 13.66 | 7.55 | 26 |
| Müll | Schlafen | Pfeife | Sack | 65 | 16.54 | 7.43 | 34 |
| Vater | Spiel | Märchen | Figur | 64 | 15.10 | 7.52 | 25 |
| Jahr | Stück | Schicht | früh | 64 | 15.56 | 7.57 | 33 |
| Schaukeln | Rolle | Dach | Stuhl | 64 | 15.26 | 8.96 | 28 |
| Start | Alarm | Schuss | Fehl | 63 | 20.55 | 7.60 | 27 |
| Krieg | Eis | Luft | Kalt | 63 | 18.60 | 7.35 | 27 |
| Wert | Alt | Korb | Papier | 63 | 17.28 | 8.68 | 27 |
| Vater | artig | Teil | groß | 62 | 16.62 | 8.67 | 26 |
| blau | Nacht | Körper | Himmel | 61 | 15.45 | 7.09 | 33 |
| Note | Bund | General | Schlüssel | 61 | 16.01 | 8.80 | 23 |
| Boot | Öl | Säge | Motor | 59 | 13.90 | 8.79 | 29 |
| Immobilie | Urteil | Moral | Wert | 59 | 23.95 | 6.44 | 29 |
| Haut | Ei | Schnee | Gans | 58 | 13.82 | 6.32 | 19 |
| Grund | Garten | Bruch | Stein | 58 | 12.17 | 6.38 | 64 |
| Licht | Geburtstag | Ständer | Kerze | 57 | 14.21 | 7.55 | 23 |
| Zeit | Kapsel | Schiff | Raum | 57 | 14.85 | 8.29 | 30 |
| Euro | Sonne | Gut | Schein | 56 | 14.55 | 8.55 | 27 |
| Kuchen | Saft | Stechen | Apfel | 55 | 20.85 | 6.27 | 22 |
| Fisch | Mine | Rausch | Gold | 55 | 16.70 | 8.88 | 29 |
| Lager | Tat | Teil | Bestand | 54 | 14.72 | 7.75 | 13 |
| Serie | Wal | Instinkt | Killer | 54 | 17.36 | 6.82 | 28 |
| Computer | Kabel | Zugriff | Netzwerk | 54 | 20.20 | 6.07 | 24 |
| Kinder | Dienst | Name | Mädchen | 52 | 10.66 | 4.76 | 25 |
| Oper | Hand | Kiste | Seife | 52 | 14.45 | 5.29 | 27 |
| Kontakt | Blick | Apfel | Auge | 50 | 18.20 | 8.36 | 28 |
| Palme | Grenze | Haus | Baum | 50 | 18.90 | 5.59 | 34 |
| Laus | Herz | Schule | Bube | 50 | 20.59 | 5.46 | 28 |
| Kammer | Maske | Erde | Gas | 50 | 14.73 | 9.19 | 24 |
| Berg | Kandidat | Partei | Spitze | 50 | 13.83 | 6.69 | 34 |
| Kleber | Star | Macht | Super | 49 | 17.80 | 7.62 | 35 |
| Liste | Sicherheit | Grenze | Kontrolle | 48 | 16.55 | 9.06 | 21 |
| Nacht | Bett | Pol | Ruhe | 48 | 19.39 | 7.50 | 23 |
| Band | Seite | Haar | Breit | 47 | 13.65 | 7.14 | 30 |
| Strecke | Pferd | Stall | Rennen | 47 | 21.73 | 8.41 | 34 |
| Sendung | Gedanke | Wort | Spiel | 47 | 15.58 | 7.31 | 30 |
| Decke | Wand | Solar | Paneel | 46 | 16.67 | 6.57 | 24 |
| Party | Tuch | Bad | Strand | 46 | 15.92 | 6.89 | 28 |
| Blumen | Geld | Los | Topf | 46 | 13.49 | 8.33 | 26 |
| Biene | Tau | Mond | Honig | 45 | 17.75 | 8.51 | 29 |
| Bahn | Zeit | Leben | Lauf | 45 | 18.55 | 6.90 | 31 |
| Linie | Patrouille | Stadt | Grenze | 44 | 16.00 | 6.81 | 25 |
| Gleichheit | Stop | Sprache | Zeichen | 44 | 18.55 | 7.26 | 25 |
| Regen | Test | Ameise | Säure | 43 | 19.17 | 5.25 | 28 |
| weise | Braut | Hufe | Paar | 42 | 19.05 | 7.61 | 24 |
| Strom | Straße | Bahn | schnell | 42 | 20.69 | 8.10 | 31 |
| Regen | Schütze | Tor | Bogen | 41 | 16.00 | 8.37 | 27 |
| Erde | Knacken | Wal | Nuss | 40 | 19.87 | 10.95 | 15 |
| Leuchten | Blei | Metall | Stift | 40 | 18.41 | 6.99 | 30 |
| Kartoffel | Holz | Wasser | süß | 40 | 20.67 | 8.31 | 30 |
| Zahn | Tomate | Sardelle | Paste | 39 | 17.08 | 6.70 | 33 |
| Zeit | Meile | Sand | Stärke | 39 | 19.37 | 7.65 | 28 |
| Kind | Art | Fach | gerecht | 38 | 15.29 | 8.42 | 26 |
| Kind | Drucken | Kaliber | klein | 38 | 15.75 | 7.31 | 26 |
| Schluss | Schrift | Frist | Kurz | 38 | 18.35 | 7.47 | 29 |
| WAHR | Brief | Lied | Liebe | 38 | 17.16 | 6.62 | 26 |
| wütig | Farbe | Schnee | blind | 36 | 14.55 | 8.67 | 39 |
| Land | Milch | Haus | Farm | 36 | 19.06 | 7.63 | 28 |
| Fänger | Kampf | Führer | Hund | 35 | 17.53 | 7.95 | 26 |
| Bad | Blume | Uhr | Sonne | 35 | 23.96 | 7.71 | 20 |
| Album | Name | Pflege | Familie | 34 | 19.40 | 7.05 | 29 |
| Schwarz | Schlag | Sommer | Loch | 34 | 17.20 | 7.76 | 29 |
| Falle | Kohle | Magen | Grube | 33 | 12.93 | 6.82 | 30 |
| Mühle | Zahn | Mehl | Säge | 33 | 17.22 | 9.45 | 24 |
| Hochzeit | Löffel | Leinwand | Silber | 33 | 20.28 | 6.41 | 27 |
| Rohr | frei | Blut | Zucker | 33 | 18.53 | 7.07 | 27 |
| Auto | Route | Gefahr | Flucht | 32 | 21.77 | 8.76 | 25 |
| Tomate | sauer | Blüte | Kirsche | 32 | 21.42 | 7.38 | 25 |
| Geschrei | Feld | Schiff | Schlacht | 32 | 20.86 | 7.11 | 25 |
| Minister | Selbst | Anwalt | Verteidigung | 32 | 16.97 | 9.71 | 28 |
| Weg | Zeile | Bar | Fuß | 29 | 23.81 | 7.04 | 35 |
| Leib | Stadt | Strand | Wache | 29 | 20.21 | 10.01 | 24 |
| Kontrolle | Ort | Rate | Geburt | 27 | 24.38 | 5.84 | 22 |
| Nebel | Nase | Haut | Horn | 26 | 18.82 | 7.36 | 27 |
| Training | Baden | Raum | Anzug | 25 | 12.20 | 6.89 | 32 |
| Mittel | Last | Führung | Beweis | 25 | 20.80 | 8.42 | 28 |
| Raub | Schutz | Privat | Kopie | 24 | 22.07 | 7.36 | 33 |
| Toilette | Staub | Reis | Schüssel | 24 | 15.88 | 11.03 | 34 |
| Kugel | Lied | Flug | Schreiber | 22 | 19.61 | 9.24 | 36 |
| Hilfe | Sparen | Daten | Paket | 21 | 17.14 | 10.88 | 39 |
| See | Bett | Wagen | Krank | 20 | 21.81 | 3.93 | 25 |
| König | Fleisch | Fang | Krabbe | 17 | 19.98 | 7.22 | 35 |
| Kind | Krank | Alt | Pflege | 17 | 15.59 | 10.66 | 23 |
| Pferd | laut | Nerv | Sport | 17 | 20.88 | 7.69 | 36 |
| Computer | Bus | Fracht | Terminal | 17 | 21.04 | 5.77 | 35 |
| Begrenzung | Spitze | Licht | Geschwindigkeit | 16 | 18.69 | 9.04 | 37 |
| Welle | Schild | Schlag | Hitze | 15 | 17.31 | 7.01 | 33 |
| Lesen | Bekenntnis | Stift | Lippe | 15 | 26.64 | 1.75 | 27 |
| Tod | Frei | Stange | Stoß | 15 | 14.73 | 9.01 | 26 |
| Brett | Kippen | Bank | Schalter | 13 | 21.35 | 4.44 | 31 |
| Branche | Land | Handel | ueblich | 10 | 13.79 | 5.65 | 29 |
| Öl | Bar | Thunfisch | Salat | 9 | 26.90 | 3.98 | 22 |
| Pistole | Auto | Einkaufen | Spielzeug | 8 | 10.98 | 13.04 | 40 |

Supplementary Table S 2. Set of compound remote associates administered inside the scanner, percentage of solved trials, mean solution times.

| Word 1 | Word 2 | Word 3 | Item | Solved% | Reaction Time Mean | Reaction Time SD | N |
| --- | --- | --- | --- | --- | --- | --- | --- |
| Park | Note | Konto | Bank | 100 | 9.52 | 7.97 | 9 |
| Spazieren | Jahr | Rund | Gang | 100 | 9.64 | 7.12 | 8 |
| Ziel | Linig | Weg | gerade | 100 | 14.48 | 4.46 | 7 |
| Biene | Tau | Mond | Honig | 100 | 29.33 | - | 1 |
| Feuer | Sommer | Platz | Lager | 100 | 18.03 | 9.67 | 9 |
| Kamerad | Lehrer | Hoch | Schule | 100 | 17.18 | 8.60 | 15 |
| Nummer | Anruf | Buch | Telefon | 100 | 9.99 | 6.99 | 15 |
| Stopp | Tasche | Turm | Uhr | 100 | 9.78 | 7.00 | 17 |
| Mittag | Wirkung | Frage | Nach | 92 | 17.90 | 7.66 | 13 |
| Traum | Licht | Anbruch | Tag | 92 | 8.90 | 6.55 | 13 |
| Boot | Öl | Säge | Motor | 91 | 16.23 | 7.80 | 11 |
| Wasser | Mine | Streuer | Salz | 91 | 8.43 | 3.57 | 11 |
| Notiz | Haus | Eis | Block | 90 | 14.43 | 9.58 | 10 |
| Wurm | Regal | Zeichen | Buch | 90 | 18.75 | 8.76 | 10 |
| Bau | Waschen | Schreiben | Maschine | 90 | 15.88 | 8.30 | 10 |
| Immer | Gras | Smaragd | Grün | 89 | 7.53 | 7.99 | 9 |
| Flocke | Mobil | Fall | Schnee | 89 | 5.77 | 2.66 | 9 |
| Tuch | Tennis | Stamm | Tisch | 89 | 11.93 | 7.68 | 9 |
| Durch | Auf | Bus | Fahrt | 87 | 19.18 | 8.99 | 15 |
| Tasche | Transfer | Strafe | Geld | 86 | 6.77 | 4.88 | 7 |
| Licht | Geburtstag | Ständer | Kerze | 86 | 12.49 | 8.39 | 14 |
| Hirn | Schlag | Mund | tot | 86 | 15.10 | 7.99 | 7 |
| Fisch | Mine | Rausch | Gold | 83 | 17.38 | 5.09 | 12 |
| Messer | Kuchen | Cottage | Käse | 83 | 8.47 | 6.21 | 12 |
| Euro | Sonne | Gut | Schein | 83 | 13.39 | 6.43 | 12 |
| Leiter | Einkauf | Last | Wagen | 83 | 9.87 | 5.12 | 6 |
| Brand | Regen | Viertel | Wald | 83 | 17.38 | 9.28 | 6 |
| Nacht | Schreibtisch | Löten | Lampe | 82 | 7.84 | 5.70 | 11 |
| Schleuse | Einfahrt | Wart | Tor | 81 | 12.86 | 8.13 | 16 |
| Kuchen | Saft | Stechen | Apfel | 80 | 20.62 | 8.36 | 10 |
| Sicherheit | Kissen | Haar | Nadel | 80 | 12.97 | 8.10 | 10 |
| Schaukeln | Rolle | Dach | Stuhl | 80 | 9.43 | 7.06 | 10 |
| Arm | Wäsche | Lage | Unter | 80 | 22.96 | 4.99 | 10 |
| Fänger | Kampf | Führer | Hund | 79 | 18.92 | 8.44 | 14 |
| Maß | Wurm | Video | Band | 78 | 13.23 | 10.78 | 9 |
| Politur | Finger | Kopf | Nagel | 78 | 18.41 | 9.38 | 9 |
| weise | Braut | Hufe | Paar | 78 | 21.71 | 9.87 | 9 |
| Müll | Schlafen | Pfeife | Sack | 78 | 10.85 | 8.34 | 9 |
| Note | Bund | General | Schlüssel | 78 | 10.99 | 5.82 | 9 |
| Berg | Kandidat | Partei | Spitze | 77 | 10.76 | 5.17 | 13 |
| Haupt | Kehrer | Laterne | Straße | 75 | 13.57 | 5.93 | 12 |
| Zahn | Haare | Schuh | Bürste | 73 | 11.17 | 7.48 | 11 |
| Bügeleisen | Schiff | Motor | Dampf | 73 | 17.12 | 7.27 | 11 |
| Zahn | Tomate | Sardelle | Paste | 73 | 13.65 | 9.20 | 11 |
| Finger | Ohr | Straße | Ring | 73 | 16.77 | 6.80 | 11 |
| Teppich | Alarmstufe | Kreuz | Rot | 73 | 12.67 | 9.28 | 11 |
| Bahn | Zeit | Leben | Lauf | 71 | 19.64 | 6.96 | 14 |
| Kleber | Star | Macht | Super | 71 | 12.93 | 12.17 | 7 |
| Regen | Schütze | Tor | Bogen | 70 | 16.52 | 9.17 | 10 |
| Start | Alarm | Schuss | Fehl | 70 | 20.07 | 6.49 | 10 |
| Kontrolle | Ort | Rate | Geburt | 70 | 17.92 | 8.45 | 10 |
| Erde | Knacken | Wal | Nuss | 70 | 17.61 | 9.92 | 10 |
| Palme | Grenze | Haus | Baum | 67 | 17.98 | 8.93 | 9 |
| Sandwich | Haus | Golf | Club | 67 | 12.70 | 9.65 | 9 |
| Lounge | Party | Kleid | Cocktail | 67 | 8.24 | 6.03 | 6 |
| Kammer | Maske | Erde | Gas | 67 | 18.76 | 8.64 | 9 |
| blau | Nacht | Körper | Himmel | 67 | 23.04 | 6.52 | 12 |
| Hart | Treiben | Fällen | Holz | 67 | 16.20 | 8.59 | 9 |
| Tomate | sauer | Blüte | Kirsche | 67 | 14.29 | 8.78 | 9 |
| Wert | Alt | Korb | Papier | 67 | 12.68 | 9.12 | 9 |
| Zeit | Kapsel | Schiff | Raum | 67 | 17.69 | 5.27 | 6 |
| Sendung | Gedanke | Wort | Spiel | 67 | 13.03 | 7.61 | 12 |
| Grund | Garten | Bruch | Stein | 65 | 13.43 | 6.11 | 20 |
| Pelz | Erde | Tasche | Mantel | 64 | 10.40 | 6.28 | 11 |
| Klick | Falle | Feld | Maus | 64 | 11.56 | 7.33 | 14 |
| Öl | Bar | Thunfisch | Salat | 64 | 14.32 | 7.58 | 11 |
| Fall | Gabe | Wahl | Aus | 63 | 20.10 | 6.82 | 8 |
| Krieg | Eis | Luft | Kalt | 63 | 12.23 | 6.61 | 8 |
| Kind | Drucken | Kaliber | klein | 63 | 9.29 | 6.95 | 8 |
| WAHR | Brief | Lied | Liebe | 63 | 14.35 | 11.55 | 8 |
| Party | Tuch | Bad | Strand | 63 | 17.90 | 9.56 | 8 |
| Bad | Blume | Uhr | Sonne | 62 | 12.59 | 7.63 | 13 |
| Lager | Tat | Teil | Bestand | 60 | 16.51 | 8.34 | 10 |
| Album | Name | Pflege | Familie | 60 | 23.44 | 4.94 | 10 |
| Pause | Bohne | Schwarz | Kaffee | 60 | 19.64 | 7.68 | 10 |
| Schwein | Fahne | Strom | Mast | 60 | 7.21 | 3.83 | 5 |
| Decke | Wand | Solar | Paneel | 60 | 13.88 | 5.97 | 10 |
| Lesen | Bekenntnis | Stift | Lippe | 56 | 14.39 | 11.78 | 9 |
| Strecke | Pferd | Stall | Rennen | 56 | 10.02 | 2.57 | 9 |
| Minister | Selbst | Anwalt | Verteidigung | 56 | 17.26 | 7.81 | 9 |
| Gleichheit | Stop | Sprache | Zeichen | 56 | 19.08 | 10.24 | 9 |
| Rohr | frei | Blut | Zucker | 56 | 16.02 | 6.07 | 9 |
| Vater | Spiel | Märchen | Figur | 55 | 6.70 | 2.03 | 11 |
| Oper | Hand | Kiste | Seife | 55 | 14.52 | 9.17 | 11 |
| Kontakt | Blick | Apfel | Auge | 54 | 17.72 | 7.50 | 13 |
| Linie | Patrouille | Stadt | Grenze | 54 | 14.61 | 8.19 | 13 |
| Vater | artig | Teil | groß | 50 | 14.89 | 7.38 | 8 |
| Falle | Kohle | Magen | Grube | 50 | 9.62 | 6.00 | 10 |
| Schwarz | Schlag | Sommer | Loch | 50 | 18.23 | 5.90 | 8 |
| Nacht | Bett | Pol | Ruhe | 50 | 13.30 | 9.43 | 12 |
| Strom | Straße | Bahn | schnell | 50 | 18.42 | 8.26 | 14 |
| Toilette | Staub | Reis | Schüssel | 50 | 15.81 | 6.35 | 12 |
| Kartoffel | Holz | Wasser | süß | 50 | 21.06 | 7.62 | 14 |
| Haut | Ei | Schnee | Gans | 45 | 18.03 | 7.54 | 11 |
| Kinder | Dienst | Name | Mädchen | 45 | 13.79 | 12.17 | 11 |
| Pferd | laut | Nerv | Sport | 45 | 19.69 | 6.21 | 11 |
| Leuchten | Blei | Metall | Stift | 45 | 13.45 | 8.45 | 11 |
| Liste | Sicherheit | Grenze | Kontrolle | 44 | 16.59 | 7.82 | 9 |
| Kind | Art | Fach | gerecht | 42 | 17.28 | 10.54 | 12 |
| Klappe | Nahrung | Pause | Baby | 40 | 20.50 | 12.22 | 5 |
| Begrenzung | Spitze | Licht | Geschwindigkeit | 40 | 19.44 | 8.05 | 10 |
| Land | Milch | Haus | Farm | 38 | 12.67 | 4.78 | 8 |
| Auto | Route | Gefahr | Flucht | 38 | 25.11 | 2.70 | 8 |
| Geschrei | Feld | Schiff | Schlacht | 38 | 18.39 | 7.53 | 8 |
| Jahr | Stück | Schicht | früh | 36 | 11.42 | 6.83 | 11 |
| Weg | Zeile | Bar | Fuß | 36 | 16.05 | 9.46 | 11 |
| Band | Seite | Haar | Breit | 33 | 11.61 | 8.66 | 9 |
| Mittel | Last | Führung | Beweis | 31 | 14.82 | 8.56 | 16 |
| Training | Baden | Raum | Anzug | 30 | 22.44 | 5.00 | 10 |
| Nebel | Nase | Haut | Horn | 30 | 9.86 | 3.06 | 10 |
| Serie | Wal | Instinkt | Killer | 30 | 17.92 | 12.09 | 10 |
| Zeit | Meile | Sand | Stärke | 29 | 20.46 | .44 | 7 |
| Tod | Frei | Stange | Stoß | 29 | 13.57 | 1.16 | 7 |
| Kugel | Lied | Flug | Schreiber | 28 | 18.75 | 5.99 | 18 |
| Laus | Herz | Schule | Bube | 25 | 20.82 | .89 | 8 |
| Schluss | Schrift | Frist | Kurz | 25 | 23.20 | 4.31 | 8 |
| Kind | Krank | Alt | Pflege | 25 | 17.55 | .89 | 12 |
| Hochzeit | Löffel | Leinwand | Silber | 25 | 22.96 | 6.22 | 12 |
| Pistole | Auto | Einkaufen | Spielzeug | 25 | 12.62 | 9.33 | 8 |
| Branche | Land | Handel | ueblich | 25 | 13.38 | 9.05 | 12 |
| Blumen | Geld | Los | Topf | 23 | 25.10 | 3.62 | 13 |
| Welle | Schild | Schlag | Hitze | 20 | 18.18 | 9.33 | 10 |
| Raub | Schutz | Privat | Kopie | 20 | 13.88 | - | 5 |
| Computer | Kabel | Zugriff | Netzwerk | 20 | 7.39 | - | 5 |
| Immobilie | Urteil | Moral | Wert | 20 | 19.37 | 11.79 | 10 |
| Hilfe | Sparen | Daten | Paket | 18 | 11.08 | 1.92 | 11 |
| Leib | Stadt | Strand | Wache | 14 | 9.22 | 2.20 | 14 |
| Computer | Bus | Fracht | Terminal | 13 | 21.16 | - | 8 |
| wütig | Farbe | Schnee | blind | 11 | 21.96 | - | 9 |
| Mühle | Zahn | Mehl | Säge | 11 | 16.64 | - | 9 |
| Brett | Kippen | Bank | Schalter | 8 | 4.31 | - | 13 |
| Punkt | Liegen | Schnitt | Wunde | 8 | 26.22 | - | 12 |
| König | Fleisch | Fang | Krabbe | 7 | 17.98 | - | 14 |
| Regen | Test | Ameise | Säure | 6 | 11.97 | - | 17 |
| See | Bett | Wagen | Krank | 0 | - | - | 10 |

Supplementary Table S 3. Mean AHA!-ratings sorted by subject and run.

| Subject | Run | Mean AHA! |
| --- | --- | --- |
| ID#02 | 1 | 2.60 |
| ID#02 | 2 | 3.75 |
| ID#02 | 3 | 3.11 |
| ID#02 | 4 | 2.83 |
| ID#03 | 1 | 2.71 |
| ID#03 | 2 | .60 |
| ID#03 | 3 | 2.50 |
| ID#03 | 4 | 3.50 |
| ID#04 | 1 | 1.88 |
| ID#04 | 2 | 1.44 |
| ID#04 | 3 | 1.20 |
| ID#04 | 4 | 1.38 |
| ID#05 | 1 | 3.00 |
| ID#05 | 2 | 4.00 |
| ID#05 | 3 | 3.33 |
| ID#05 | 4 | 3.60 |
| ID#06 | 1 | 2.44 |
| ID#06 | 2 | 3.00 |
| ID#06 | 3 | 1.25 |
| ID#06 | 4 | .78 |
| ID#07 | 1 | 3.75 |
| ID#07 | 2 | 2.50 |
| ID#07 | 3 | 4.33 |
| ID#07 | 4 | 4.29 |
| ID#08 | 1 | 4.00 |
| ID#08 | 2 | 4.13 |
| ID#08 | 3 | 4.30 |
| ID#08 | 4 | 4.25 |
| ID#09 | 1 | 3.00 |
| ID#09 | 2 | 3.43 |
| ID#09 | 3 | 3.00 |
| ID#09 | 4 | 3.00 |
| ID#10 | 1 | 4.00 |
| ID#10 | 2 | 3.50 |
| ID#10 | 3 | 2.91 |
| ID#10 | 4 | 3.25 |
| ID#11 | 1 | 2.38 |
| ID#11 | 2 | 2.29 |
| ID#11 | 3 | 2.63 |
| ID#11 | 4 | 3.00 |
| ID#12 | 1 | 2.00 |
| ID#12 | 2 | 1.71 |
| ID#12 | 3 | 2.11 |
| ID#12 | 4 | 1.75 |
| ID#13 | 1 | 3.40 |
| ID#13 | 2 | 3.25 |
| ID#13 | 3 | 3.38 |
| ID#13 | 4 | 4.00 |
| ID#14 | 1 | 2.80 |
| ID#14 | 2 | 4.00 |
| ID#14 | 3 | 4.25 |
| ID#14 | 4 | 4.50 |
| ID#15 | 1 | 2.00 |
| ID#15 | 2 | 2.22 |
| ID#15 | 3 | 2.63 |
| ID#15 | 4 | 3.33 |
| ID#16 | 1 | 2.45 |
| ID#16 | 2 | 2.40 |
| ID#16 | 3 | 2.30 |
| ID#16 | 4 | 3.00 |
| ID#17 | 1 | 2.38 |
| ID#17 | 2 | 2.71 |
| ID#17 | 3 | 2.40 |
| ID#17 | 4 | 2.60 |
| ID#18 | 1 | 3.88 |
| ID#18 | 2 | 3.90 |
| ID#18 | 3 | 3.27 |
| ID#18 | 4 | 3.40 |
| ID#19 | 1 | 1.25 |
| ID#19 | 2 | 1.00 |
| ID#19 | 3 | .89 |
| ID#19 | 4 | 1.50 |
| ID#20 | 1 | 2.11 |
| ID#20 | 2 | 3.57 |
| ID#20 | 3 | 4.00 |
| ID#20 | 4 | 4.13 |
| ID#21 | 1 | 1.88 |
| ID#21 | 2 | 2.50 |
| ID#21 | 3 | 3.00 |
| ID#21 | 4 | 2.50 |
| ID#22 | 1 | 2.67 |
| ID#22 | 2 | 2.11 |
| ID#22 | 3 | 2.50 |
| ID#22 | 4 | 3.00 |
| ID#23 | 1 | 2.20 |
| ID#23 | 2 | 2.20 |
| ID#23 | 3 | 1.67 |
| ID#23 | 4 | 1.63 |
| ID#24 | 1 | 4.00 |
| ID#24 | 2 | 3.17 |
| ID#24 | 3 | 4.60 |
| ID#24 | 4 | 4.17 |
| ID#25 | 1 | 1.43 |
| ID#25 | 2 | 3.00 |
| ID#25 | 3 | 3.88 |
| ID#25 | 4 | 3.83 |
| ID#26 | 1 | 2.57 |
| ID#26 | 2 | 2.00 |
| ID#26 | 3 | 3.50 |
| ID#26 | 4 | 3.00 |
| ID#27 | 1 | 1.75 |
| ID#27 | 2 | 3.00 |
| ID#27 | 3 | 2.00 |
| ID#27 | 4 | 1.57 |
| ID#28 | 1 | 1.13 |
| ID#28 | 2 | 2.33 |
| ID#28 | 3 | 2.00 |
| ID#28 | 4 | 2.75 |
| ID#29 | 1 | 3.60 |
| ID#29 | 2 | 2.20 |
| ID#29 | 3 | 4.00 |
| ID#29 | 4 | 4.43 |
| ID#30 | 1 | 3.50 |
| ID#30 | 2 | 4.00 |
| ID#30 | 3 | 3.36 |
| ID#30 | 4 | 3.25 |
